# Huanglian Wendan decoction modulates gut microbiota-brain axis for depression comorbid with insomnia: a mini review

**DOI:** 10.3389/fmicb.2026.1841363

**Published:** 2026-07-15

**Authors:** Yanhong Ban, Qingchun Shi, Yan Li, Limin Pan, Ruiqian Guan

**Affiliations:** 1 Department of Traditional Chinese Medicine, The Second Affiliated Hospital of Heilongjiang University of Chinese Medicine, Harbin, China; 2 Department of Traditional Chinese Medicine, Baotou Mongolian Medicine and Chinese Medicine Hospital, Baotou, China; 3 Department of Traditional Chinese Medicine, Qingdao Eighth People’s Hospital, Qingdao, China; 4 Department of Traditional Chinese Medicine, The First Affiliated Hospital of Heilongjiang University of Chinese Medicine, Harbin, China; 5 Department of Acupuncture and Massage, The Second Affiliated Hospital of Heilongjiang University of Chinese Medicine, Harbin, China

**Keywords:** depression comorbid with insomnia, gut microbiota, Huanglian Wendan Decoction, intestinal mucosal barrier, microbiota-gut-brain axis, short-chain fatty acids

## Abstract

Depression comorbid with insomnia (DCI) is a highly prevalent mental disorder with complex pathogenesis, posing great clinical challenges for Western medical interventions due to adverse reactions, drug dependence and single targets. Accumulating preclinical evidence confirms the microbiota-gut-brain axis (MGBA) as a core regulatory hub in DCI pathogenesis, with gut microbiota dysbiosis being the initiating factor that disrupts host neuroendocrine, immune and circadian systems via multiple pathways, forming a vicious circle to aggravate DCI symptoms. Huanglian Wendan Decoction (HLWDD), a classic traditional Chinese medicine herbal formula, shows potential therapeutic effects on DCI by targeting the gut microbiota—the core node of MGBA. Notably, most relevant evidence is derived from animal preclinical models. This Mini Review succinctly summarizes the core pathological mechanisms of gut microbiota dysbiosis inducing DCI via MGBA, and elaborates HLWDD’s multi-target therapeutic effects on DCI centered on gut microbiota regulation: reshaping gut microbiota composition, promoting short-chain fatty acids (SCFAs) production, and protecting intestinal mucosal barrier integrity, as well as improving central nervous system dysfunction via MGBA bidirectional regulation. We also discuss the existing academic controversies, critical research gaps and potential future development directions in this field. This review highlights the gut microbiota as a key therapeutic target of HLWDD for DCI, and provides a scientific basis for its clinical application and translational research via MGBA modulation.

## 1 Introduction

Depression comorbid with insomnia (DCI) is a highly prevalent and burdensome mental comorbidity worldwide. Insomnia involves persistent sleep dissatisfaction with functional impairment, while depression features prolonged low mood and anhedonia with social and daily life impacts (GBD 2017 Disease and Injury Incidence and Prevalence Collaborators, 2018). Its prevalence has risen markedly over decades and surged further due to the COVID-19 pandemic (GBD 2017 Disease and Injury Incidence and Prevalence Collaborators, 2018; Pappa et al., 2020); over 70% of depression patients have sleep disturbances, and sleep disorders raise depression risk 3–4 times (Chinese Society of Neurology, Chinese Society of Sleep Disorders, and Chinese Society of Neuropsychology and Behavioral Neurology, 2020). The two conditions form a bidirectional causal relationship, with sleep disorders acting as an independent risk factor for depression onset and recurrence across all ages (Fang et al., 2019), leading to severe clinical manifestations, low remission rates and high recurrence risks. Western pharmacological interventions for DCI face inherent limitations: antidepressants may impair sleep quality, while hypnotics carry risks of dependence and may exacerbate depressive symptoms (Doghramji and Jangro, 2016), and non-pharmacological therapies are restricted by poor patient adherence (Asarnow and Manber, 2019). In recent years, the microbiota-gut-brain axis (MGBA) has emerged as a pivotal regulatory pathway in DCI pathogenesis, with gut microbiota being the core mediator linking the gut and the central nervous system (CNS) (Bathgate et al., 2017; Riehl et al., 2024). The gut microbiota maintains host emotional state and sleep-wake cycle homeostasis by releasing SCFAs, tryptophan metabolites and neurotransmitters (Chang et al., 2022; Riehl et al., 2024), while gut microbiota dysbiosis (reduced diversity, imbalanced flora structure, decreased beneficial bacteria and overgrowth of pathogenic bacteria) disrupts MGBA function, initiating and aggravating DCI (Radjabzadeh et al., 2022; Reyes-Martínez et al., 2023). A large number of preclinical animal studies and preliminary clinical observations have confirmed obvious structural and functional abnormalities of gut microbiota in DCI, with significantly reduced SCFAs levels (Liu et al., 2022; Pearson-Leary et al., 2020), indicating that gut microbiota is a potential therapeutic target for DCI.

Traditional Chinese medicine (TCM) has unique advantages in DCI treatment due to its multi-target, multi-pathway effects and good safety profile (Che et al., 2022). Huanglian Wendan Decoction (HLWDD), a classic TCM formula, has been widely used in clinical DCI treatment, andpreclinical animal studies have verified its efficacy in improving sleep and depressive symptoms by regulating the MGBA (Shi et al., 2025). Notably, HLWDD exerts its therapeutic effects by taking gut microbiota regulation as the core starting point, and its active components can synergistically reshape gut microbiota structure, restore intestinal mucosal barrier and promote the production of microbial metabolites such as SCFAs (Liu et al., 2025; Shi et al., 2024). Despite the promising preclinical findings, there are still academic controversies about the core regulatory targets of HLWDD on gut microbiota and the key pathological features of DCI-related gut microbiota dysbiosis, as well as critical research gaps in clinical translation and molecular mechanism elucidation. Based on the MGBA theory, this Mini Review focuses on the gut microbiota as the core node, systematically summarizes the pathological mechanism of gut microbiota dysbiosis inducing DCI and the therapeutic mechanism of HLWDD regulating gut microbiota to improve DCI, discusses the existing controversies and research gaps, and prospects future research directions, aiming to provide a focused and up-to-date reference for the gut microbiota-centered translational research of HLWDD in DCI treatment.

## 2 Gut microbiota dysbiosis: the core initiator of MGBA dysfunction in DCI

As the core component of the MGBA, the gut microbiota and its metabolites are essential signaling mediators regulating CNS function and sleep-wake cycles, and gut microbiota dysbiosis is the key pathological link in the occurrence and development of DCI (Matenchuk et al., 2020; Reyes-Martínez et al., 2023). DCI exhibit significant gut microbiota structural and functional abnormalities, which form a vicious circle with depressive and insomnia symptoms via the MGBA. Notably, most mechanistic insights into the MGBA are derived from animal models; human evidence remains limited, and interspecies differences should be considered when extrapolating findings to clinical DCI.

**FIGURE 1:**
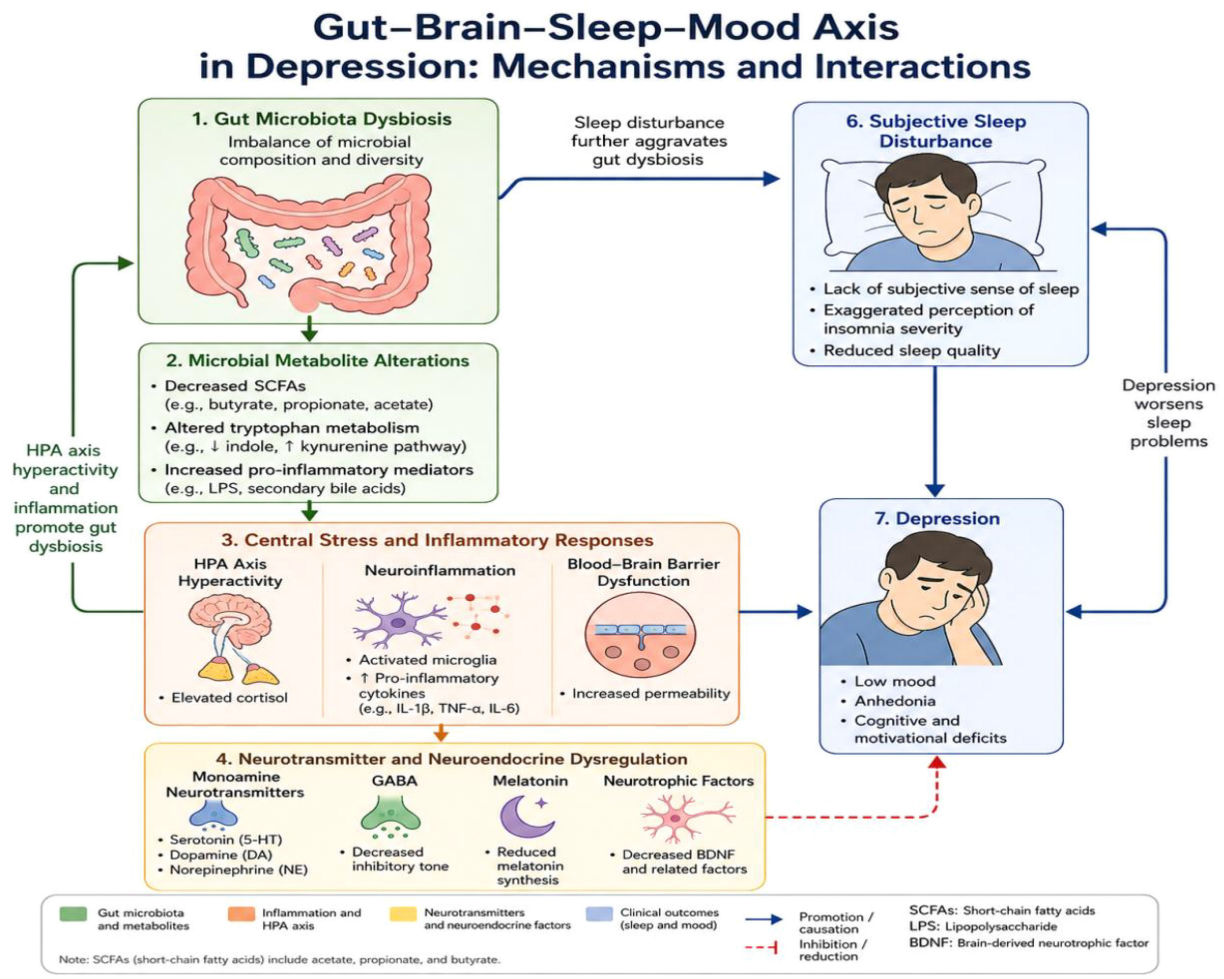
Schematic overview of the bidirectional interactions among depression, sleep disturbance, and gut microbiota dysbiosis. This schematic illustrates the gut-brain-sleep-mood axis in depression, depicting a bidirectional, self-amplifying cycle linking gut microbiota dysbiosis, central inflammatory and stress responses, neurotransmitter dysregulation, subjective sleep disturbances, and clinical depression. Gut microbiota dysbiosis, defined by altered microbial composition and diversity, drives microbial metabolite alterations, including reduced short-chain fatty acids (SCFAs), disrupted tryptophan metabolism, and elevated pro-inflammatory mediators (LPS, secondary bile acids). These metabolic perturbations trigger central stress and inflammatory responses, characterized by HPA axis hyperactivity (elevated cortisol), neuroinflammation (activated microglia and increased pro-inflammatory cytokines such as IL-1β, TNF-α, and IL-6), and blood-brain barrier dysfunction (increased permeability). This cascade leads to neurotransmitter and neuroendocrine dysregulation, including imbalanced monoamine neurotransmitters (serotonin, dopamine, norepinephrine), reduced GABAergic inhibitory tone, impaired melatonin synthesis, and decreased neurotrophic factors (BDNF). These biological abnormalities manifest clinically as subjective sleep disturbances (poor sleep quality, exaggerated insomnia perception, and reduced sense of sleep) and depressive symptoms (low mood, anhedonia, and cognitive/motivational deficits). Critically, reciprocal interactions sustain the vicious cycle: sleep disturbances further aggravate gut dysbiosis, while depression exacerbates sleep problems and inhibits neurotrophic factor production, perpetuating the pathological loop.

The pathological mechanisms by which gut microbiota dysbiosis induces DCI via the MGBA are closely related to the regulation of gut microbiota on host neurotransmitter, neuroendocrine, immune, intestinal barrier and circadian systems, with all pathways centered on gut microbiota dysbiosis and mutually promoting each other. The above bidirectional pathological cycle is intuitively illustrated in Figure 1.

### 2.1 Disrupting central neurotransmitter homeostasis via microbial metabolism

The gut microbiota is the main site for host neurotransmitter synthesis, participating in the production of more than 90% of serotonin (5-HT), a large amount of γ-aminobutyric acid (GABA) and dopamine (DA) (Breit et al., 2018), these transmitters are critical for emotional and sleep regulation. Gut microbiota dysbiosis reduces the synthesis of these inhibitory neurotransmitters and increases the level of excitatory neurotransmitter glutamic acid (Glu), leading to the imbalance of CNS excitatory-inhibitory neurotransmitter system (elevated Glu/GABA ratio) (Zhong et al., 2019). Meanwhile, the reduction of SCFAs in DCI further impairs the reuptake and metabolism of central neurotransmitters (Socała et al., 2021), directly disrupting the normal function of hippocampus, prefrontal cortex and suprachiasmatic nucleus, and inducing DCI. Microbial 5-HT and GABA are highly hydrophilic and barely cross the intact BBB, so they cannot directly enter the brain (Chen et al., 2021). These neurotransmitters regulate central functions indirectly via intestinal receptors, peripheral nerves and the vagus afferent pathway. By contrast, microbial metabolites such as SCFAs and tryptophan derivatives penetrate the BBB and maintain central neurotransmitter homeostasis, acting as key mediators of gut-brain communication (Zhuang et al., 2024).

### 2.2 Inducing HPA axis overactivation via intestinal barrier damage

Hyperexcitability of the HPA axis is a shared pathological feature of DCI (Wang H. et al., 2022), and gut microbiota dysbiosis is an important trigger for HPA axis overactivation. Dysbiosis leads to decreased beneficial bacteria and reduced SCFAs production, damaging the intestinal mucosal barrier and increasing intestinal permeability (Liu et al., 2023). Pathogenic bacterial lipopolysaccharide (LPS) enters the blood circulation to form metabolic endotoxemia, activating the immune system and releasing pro-inflammatory factors (Hashimoto, 2023). These factors act on the hypothalamus and pituitary gland, causing excessive secretion of corticotropin releasing hormone (CRH) and adrenocorticotropic hormone (ACTH), and ultimately excessive cortisol synthesis and release. High cortisol levels disrupt circadian rhythm, impair non-REM sleep quality, and enhance the brain’s susceptibility to negative emotions (Liu et al., 2015; Vgontzas and Chrousos, 2002), while HPA axis overactivation further damages the intestinal mucosal barrier, forming a gut microbiota dysbiosis-intestinal barrier damage-HPA axis overactivation-DCI vicious circle.

### 2.3 Triggering neuroinflammation via microbial-immune crosstalk

Gut microbiota dysbiosis is the core cause of chronic low-grade inflammation in DCI, and neuroinflammation is the key bridge linking gut microbiota dysbiosis and CNS damage (Reyes-Martínez et al., 2023). On the one hand, dysbiosis-induced intestinal barrier damage leads to LPS and peptidoglycan entering the systemic circulation, activating the toll-like receptor 4 (TLR4)/NF-κB signaling pathway and releasing a large number of pro-inflammatory factors (TNF-α, IL-6, IL-1β) in peripheral blood (Góralczyk-Bińkowska et al., 2022). On the other hand, these pro-inflammatory factors cross the blood-brain barrier (BBB) or enter the brain via the vagus nerve, activating microglia and astrocytes, and inducing central neuroinflammation (Troubat et al., 2021). In addition, the reduction of SCFAs leads to excessive polarization of microglia to the M1 pro-inflammatory phenotype (Huang et al., 2023), further exacerbating central neuroinflammation, which in turn causes neuronal damage and synaptic plasticity impairment, and disrupts neurotransmitter and HPA axis function, aggravating DCI.

### 2.4 Disrupting host circadian rhythm via microbial circadian disorder

The gut microbiota has an independent circadian rhythm, with up to 60% of its composition showing rhythmic fluctuations consistent with the host’s circadian cycle (Song et al., 2021), and the dominant phyla Bacteroidetes and Firmicutes exhibit distinct diurnal cycling patterns closely related to the host’s sleep-wake cycle (Li Y. et al., 2018). The gut microbiota regulates host circadian rhythm mainly by producing SCFAs, melatonin and tryptophan metabolites, which act on the suprachiasmatic nucleus and regulate the expression of clock genes (CLOCK, BMAL1, PER, CRY) (Seong et al., 2024), and by regulating gastrointestinal hormone secretion. Gut microbiota dysbiosis disrupts its own circadian rhythm, leading to host circadian clock disorder, sleep-wake cycle disturbance and hormone secretion imbalance (Matenchuk et al., 2020), while host sleep disturbance further aggravates gut microbiota dysbiosis, forming a bidirectional regulatory disorder between gut microbiota and host circadian rhythm.

## 3 HLWDD regulates gut microbiota to ameliorate DCI via MGBA

**FIGURE 2:**
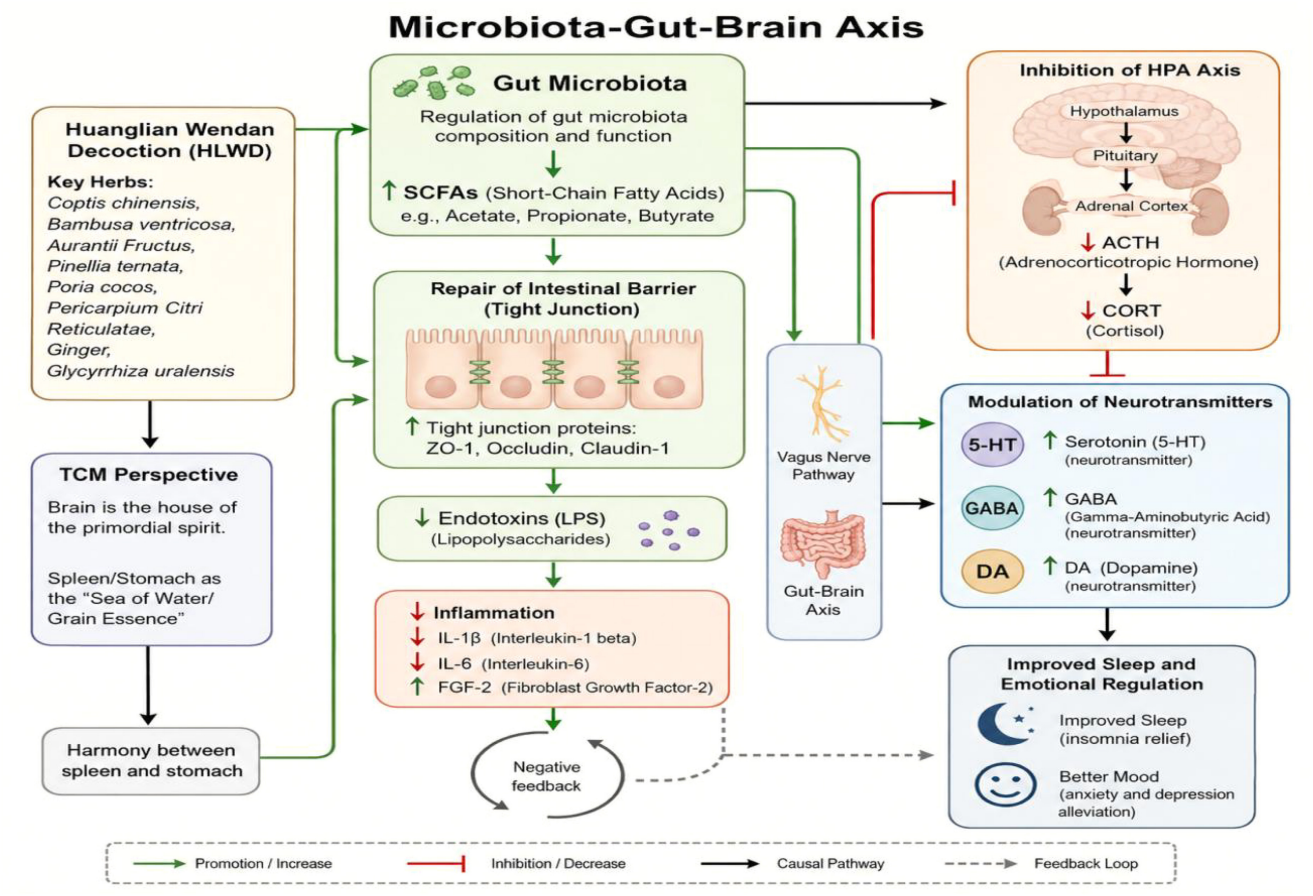
Mechanistic diagram of HLWDD in modulating the MGBA for DCI. This schematic illustrates the mechanisms by which Huanglian Wendan Decoction (HLWD) regulates the microbiota-gut-brain axis to improve sleep and emotional regulation. From a TCM perspective, HLWD exerts therapeutic effects by harmonizing the spleen and stomach, thereby influencing the brain, the “house of the primordial spirit.” Pharmacologically, HLWD modulates gut microbiota composition and function, promoting the production of SCFAs such as acetate, propionate, and butyrate. These SCFAs facilitate the repair of the intestinal barrier by upregulating tight junction proteins (ZO-1, Occludin, Claudin-1), reducing the translocation of endotoxins (LPS) and inhibiting systemic inflammation via decreased IL-1β, IL-6, and increased FGF-2. This anti-inflammatory effect, in turn, forms a negative feedback loop. Concurrently, HLWD inhibits the HPA axis, lowering ACTH and cortisol levels, and modulates neurotransmitters including 5-HT, GABA, and DA through the vagus nerve pathway. Collectively, these actions lead to improved sleep (insomnia relief) and better mood (anxiety and depression alleviation).

HLWDD exerts a comprehensive therapeutic effect on DCI by taking gut microbiota regulation as the core starting point, restoring MGBA homeostasis through multi-target and multi-pathway mechanisms, which is consistent with the multi-component synergistic characteristics of TCM formulas and the complex pathogenesis of DCI (Table 1). Each medicinal component of HLWDD has a specific regulatory effect on the gut microbiota, and the whole formula exerts a synergistic effect to reshape gut microbiota structure, protect intestinal mucosal barrier and promote beneficial metabolite production, and further regulate the central nervous system through the MGBA bidirectional communication, thus alleviating DCI symptoms from peripheral to central. All mechanisms summarized herein are supported by preclinical animal studies; clinical evidence in humans regarding efficacy and pharmacokinetic profiles remains limited. The overall therapeutic regulatory network of HLWDD targeting the microbiota-gut-brain axis for DCI is visualized in Figure 2.

**TABLE 1:** Active ingredients, sources, mechanisms and models of HLWDD for DCI via MGBA.

Active ingredient	Main effects	Signaling pathways / mechanisms	References
Berberine	In animal models of DCI and intestinal injury: Increase beneficial bacteria; inhibit pathogenic bacteria; protect intestinal barrier; suppress neuroinflammation	Upregulate tight junction proteins (Claudin-1, Occludin, ZO-1); inhibit TLR4/NF-κB/NLRP3 pathway	Jia et al., 2018; Zhou et al., 2023
Polysaccharides	In animal insomnia models and *in vitro* microbial models: Promote proliferation of Lactobacillus and Bifidobacterium; elevate SCFAs; enhance gut microbiota diversity	Act as prebiotics; promote microbial fermentation of dietary fiber	Gao et al., 2024; Huang et al., 2019; Li C. J. et al., 2018
Total flavonoids	In animal models of intestinal inflammation and insomnia: Alleviate intestinal inflammation; prevent gut microbiota translocation; regulate neurotransmitters	Inhibit JAK1/STAT3 pathway; adjust Glu/GABA ratio	Chen et al., 2022; Jia et al., 2016; Li et al., 2023
Hesperidin	In chronic stress depression animal models: Inhibit HPA axis overactivation; reduce cortisol level	Upregulate glucocorticoid receptor expression	Cai et al., 2013; Zhou et al., 2018
Pachyman	In antibiotic-induced gut dysbiosis animal models: Enhance gut microbiota diversity; maintain intestinal homeostasis	Regulate intestinal biological barrier function	Li C. J. et al., 2018

### 3.1 Reshaping gut microbiota composition and promoting SCFAs production

Regulating gut microbiota structure and restoring its functional homeostasis is the core mechanism of HLWDD for DCI treatment (Shi et al., 2024). The active components of HLWDD (flavonoids, chalcones, phenols, polysaccharides) play a key role in gut microbiota regulation, with each herb exerting a specific synergistic effect: Coptis chinensisBerberine is recognized as a major active contributor from within the multi-component therapeutic framework of HLWDD. In animal models, polysaccharides and berberine from Coptis chinensis modulate intestinal tight junction protein expression, increase the abundance of beneficial Lactobacillus, and suppress the proliferation of pathogenic Bacteroides Acidifaciens (Wang Y. E. et al., 2022; Zhou et al., 2023); Pinelliae Rhizoma (Banxia) and Bambusae Caulis in Taeniam (Zhuru) promote the proliferation of Bifidobacterium and Bacteroides through their polysaccharides *in vivo* (Gao et al., 2024; Huang et al., 2019); Aurantii Fructus Immaturus (Zhishi) alleviates intestinal inflammation and prevents gut microbiota translocation via its total flavonoids in animal models (Chen et al., 2022); Poria cocos (Fuling) enhances gut microbiota diversity and maintains intestinal homeostasis (Li C. J. et al., 2018). Preclinical studies have confirmed that HLWDD intervention can significantly restore the abundance and diversity of fecal microbial communities in insomnia model rats, increase the abundance of Lactobacillus and Bifidobacterium, and reduce pathogenic bacteria such as Desulfovibrio (Shi et al., 2024), partially reverse DCI-related gut microbiota dysbiosis.

SCFAs—the main metabolite of gut microbiota fermenting dietary fiber—are the key signal molecules for HLWDD to regulate the MGBA (Mansuy-Aubert and Ravussin, 2023; Socała et al., 2021). In DCI animal models, HLWDD significantly promotes the synthesis and secretion of SCFAs (acetic acid, propionic acid, butyric acid) by the gut microbiota (Shi et al., 2024; Shi et al., 2025): its polysaccharides and flavonoids act as prebiotics for beneficial bacteria, promoting their fermentation and thus increasing fecal and serum SCFAs levels, and the elevation of SCFAs levels is positively correlated with the improvement of DCI symptoms (Shi et al., 2024; Zhang et al., 2024). SCFAs exert multiple beneficial effects on DCI, including anti-inflammation, neurotransmitter regulation, HPA axis inhibition and circadian rhythm modulation, and are the important peripheral mediators for HLWDD to regulate the MGBA (Burokas et al., 2017; Wang et al., 2025).

### 3.2 Protecting intestinal mucosal barrier to block MGBA dysfunction vicious circle

The integrity of the intestinal mucosal barrier is the basis for maintaining normal MGBA function, and protecting the intestinal mucosal barrier is an important peripheral link for HLWDD to treat DCI (Cui et al., 2026). HLWDD protects the intestinal mucosal barrier and reduces intestinal permeability through three synergistic pathways, centered on gut microbiota regulation: first, it enhances the intestinal mechanical barrier by upregulating the expression of tight junction proteins (Claudin 1, Occludin, ZO-1) via berberine, polysaccharides and total flavonoids, inhibiting intestinal epithelial cell apoptosis in animal models (Zhou et al., 2023; Zhou et al., 2025); second, it stabilizes the intestinal biological barrier by reshaping gut microbiota structure, making beneficial bacteria form a dominant flora on the intestinal mucosal surface and preventing pathogenic bacteria adhesion and colonization (Shi et al., 2025; Yu et al., 2025); third, it regulates the intestinal immune barrier by inhibiting intestinal inflammation, reducing pro-inflammatory factor levels and maintaining immune system balance (Yi, 2023; Zhu et al., 2025). The protection of the intestinal mucosal barrier by HLWDD effectively prevents leaky gut and metabolic endotoxemia, blocks the pathological pathway of gut microbiota dysbiosis inducing peripheral and central inflammation, and breaks the vicious circle of gut microbiota dysbiosis-MGBA dysfunction-DCI.

### 3.3 Regulating central nervous system function via MGBA bidirectional communication

Based on gut microbiota regulation and intestinal barrier protection, HLWDD further modulates CNS function through the MGBA bidirectional signaling pathway, reversing the central pathological changes of DCI, and these central regulatory effects are all mediated by gut microbiota and its metabolites. HLWDD promotes the synthesis of 5-HT in the gut and brain by regulating gut microbiota and SCFAs production, increases the content of 5-HT and GABA in the hippocampus and prefrontal cortex, and reduces the level of Glu, thus restoring the excitatory-inhibitory balance of the CNS neurotransmitter system (Hu et al., 2022; Li et al., 2023). Animal studies have confirmed that HLWDD can adjust the Glu/GABA ratio to normal levels and regulate DA and norepinephrine levels, effectively improving DCI-related neural regulation imbalance (Li et al., 2023). HLWDD inhibits the activation of NF-κB and JAK1/STAT3 signaling pathways in the peripheral and central nervous systems via its flavonoids and berberine, reducing the release of pro-inflammatory factors (TNF-α, IL-6) (Jia et al., 2016; Jia et al., 2018), and promoting the polarization of microglia from M1 pro-inflammatory phenotype to M2 anti-inflammatory phenotype (Su et al., 2025; Zheng et al., 2023). Meanwhile, SCFAs produced by gut microbiota after HLWDD intervention further inhibit microglia activation and neuroinflammation (Huang et al., 2023; Shi et al., 2024). By suppressing neuroinflammation, HLWDD upregulates the expression of brain-derived neurotrophic factor (BDNF) and its receptor TrkB in the hippocampus, promotes neuronal survival and proliferation, and enhances synaptic plasticity (Shi et al., 2025; Wu et al., 2025), thus repairing CNS structural and functional damage in DCI.

In the next place, HLWDD inhibits HPA axis overactivation and restores its negative feedback regulation mechanism through direct and indirect pathways: its active components (hesperidin, Aurantii fructus alcohol) directly upregulate glucocorticoid receptor expression and reduce serum cortisol levels (Cai et al., 2013; Zhou et al., 2018); indirectly, it blocks the activation of HPA axis by pro-inflammatory factors through inhibiting chronic inflammation (Hashimoto, 2023; Jia et al., 2018), and SCFAs act on the HPA axis via the vagus nerve to inhibit its overactivation (Socała et al., 2021). The normalization of HPA axis function further protects the intestinal mucosal barrier and regulates gut microbiota, forming a positive cycle of MGBA homeostasis restoration. HLWDD restores the circadian rhythm of dominant gut microbiota phyla (Bacteroidetes, Firmicutes) by reshaping gut microbiota composition, and SCFAs produced by gut microbiota act on the suprachiasmatic nucleus to regulate the expression of core clock genes, restoring the host’s circadian clock (Sang et al., 2025; Seong et al., 2024; Song et al., 2021). Meanwhile, HLWDD increases brain GABA and melatonin levels and restores cortisol secretion circadian rhythm by regulating the neurotransmitter system and HPA axis (Hu et al., 2022; Liu et al., 2015), thus improving sleep-wake cycle disorder in DCI.

## 4 Discussion

Given the vital contribution of gut microbiota dysbiosis to the progression of DCI, this review systematically elaborates the mechanisms through which HLWDD alleviates DCI by remodeling intestinal microecology and maintaining MGBA homeostasis. Accumulating preclinical findings suggest that gut microbiota serves as a central therapeutic target of HLWDD, enabling the formula to exert multi-modal therapeutic effects via modulating gut microbiota and optimizing MGBA function. Nevertheless, current studies still present several unresolved controversies, ambiguous molecular mechanisms, and limited translational evidence, which hinder the in-depth mechanistic exploration and clinical application of HLWDD. Notably, existing supportive evidence is primarily obtained from animal and *in vitro* experiments. Considering inherent interspecies differences, the low bioavailability of herbal active ingredients, and the lack of sufficient clinical validation, preclinical observations cannot be simply generalized to human conditions. Accordingly, the interpretation and clinical transformation of relevant findings should be treated with caution. Combined with recent research advances, this section further discusses core academic controversies, key research limitations, and potential future research directions in this field.

Two key academic controversies currently exist regarding HLWDD-mediated gut microbiota regulation for DCI treatment. First, no unified conclusion has been reached on the core microbial pathological characteristics of DCI. Several studies regard the imbalance of the Firmicutes/Bacteroidetes ratio as the dominant feature of DCI-associated dysbiosis, whereas multiple recent studies suggest that the decreased abundance of SCFA-producing beneficial bacteria (Lactobacillus and Bifidobacterium) and the overgrowth of pro-inflammatory pathogenic bacteria constitute the primary intestinal pathological alterations in DCI. Such inconsistent results may be attributable to differences in experimental models, detection techniques, and individual heterogeneity of research subjects.

Second, the conflicting viewpoints regarding single-component efficacy versus whole-formula synergism represent the central unresolved controversy in HLWDD research. Several studies regard berberine as the major effective constituent responsible for gut microbiota regulation, while mainstream pharmacological studies support that the synergistic interactions of multiple active ingredients, including alkaloids, polysaccharides, and flavonoids, collectively contribute to the microecological regulatory efficacy of HLWDD. Of note, no controlled study has so far directly compared the therapeutic effects of isolated berberine and intact HLWDD intervention under identical DCI experimental conditions. Whether berberine alone can fully recapitulate the comprehensive gut-regulatory and DCI-ameliorating effects of the complete formula remains unvalidated. This dilemma reflects the holistic compatibility characteristics of traditional Chinese medicine formulas and indicates that single-component pharmacological research is insufficient to interpret the overall therapeutic advantages of HLWDD.

Pharmacokinetic and pharmacological evidence further explains such component differences. Berberine possesses extremely low intestinal absorption and systemic bioavailability, thereby exerting only local intestinal microecological regulation rather than systemic central neuro-modulation. In contrast, the polysaccharide components of HLWDD exhibit prominent prebiotic properties, nourishing beneficial gut microbiota, promoting SCFA biosynthesis and metabolism, and further amplifying MGBA regulatory effects. The complementary actions between poorly absorbed alkaloids and prebiotic polysaccharides form the irreplaceable multi-component synergistic superiority of the complete HLWDD formula.

Although preclinical studies have demonstrated the promising efficacy of HLWDD against DCI, multiple research gaps severely restrict its clinical translation. First, the overall evidence level remains low. Current studies are dominated by small-sample animal experiments, lacking large-scale, multicenter, randomized, double-blind, long-term controlled clinical trials. The clinical efficacy, safety, and long-term adverse reactions of HLWDD in DCI have not been systematically validated. The correlation between gut microbiota improvement and clinical symptom remission remains unclear. Moreover, clinical data regarding the safety and efficacy of HLWDD monotherapy or combined administration with clinical antidepressants and hypnotics are extremely scarce, restricting its standardized clinical application. Second, the core molecular targets, key signaling pathways, and multi-pathway crosstalk networks of HLWDD in regulating intestinal microecology and MGBA function remain poorly clarified. The interaction patterns between active components and gut microbiota, as well as the upstream and downstream metabolic regulatory mechanisms, require further systematic verification. Third, population-based individualized research is lacking. Existing studies have not stratified DCI according to age, gender, or TCM syndrome types, and no personalized microecological intervention strategy for HLWDD has been established. Fourth, the *in vivo* metabolic characteristics of HLWDD active components remain unclear. The absorption, distribution, and metabolic profiles of multiple HLWDD constituents under DCI pathological conditions are still undefined. Meanwhile, the potential drug–drug interaction risks between HLWDD and commonly used psychotropic Western drugs have not been explored, limiting the safe and rational clinical combination medication.

To address the aforementioned limitations and advance the clinical translation of HLWDD, future studies are warranted to conduct systematic and targeted research. First, well-designed component modification experiments may help clarify the independent efficacy of major active ingredients and elucidate the synergistic interactions among multiple components, which is conducive to resolving the ongoing debate regarding single-component versus whole-formula efficacy. Second, large-scale, multicenter clinical trials incorporating gut microbiota profiles and SCFA levels as core biomarkers are needed to validate the clinical efficacy and safety of HLWDD and clarify the correlation between microecological improvement and DCI symptom remission. Third, establishing a standardized safety assessment system covering adverse reaction surveillance, toxicological detection and drug interaction evaluation can complement the insufficient clinical safety data of HLWDD. Fourth, population stratification studies based on gut microbial characteristics and TCM syndrome differentiation may reveal the heterogeneous regulatory effects of HLWDD, supporting its individualized and precise clinical application. Fifth, further exploration of HLWDD-mediated modulation of gut microbial circadian rhythms will deepen our understanding of its mechanism in ameliorating DCI-related circadian disorders and enrich the theoretical basis for HLWDD to restore MGBA homeostasis and treat DCI.
